# Chemokine Pathways in Cutaneous Melanoma: Their Modulation by Cancer and Exploitation by the Clinician

**DOI:** 10.3390/cancers13225625

**Published:** 2021-11-10

**Authors:** Rebecca Adams, Bernhard Moser, Sophia N. Karagiannis, Katie E. Lacy

**Affiliations:** 1 St. John’s Institute of Dermatology, School of Basic & Medical Biosciences, King’s College London, London WC2R 2LS, UK; rebecca.1.adams@kcl.ac.uk; 2 Division of Infection & Immunity, Henry Wellcome Building, Cardiff University School of Medicine, Heath Park, Cardiff CF14 4YS, UK; moserb@cardiff.ac.uk; 3 Guy’s Cancer Centre, Breast Cancer Now Research Unit, School of Cancer & Pharmaceutical Sciences, King’s College London, London WC2R 2LS, UK

**Keywords:** chemokines, melanoma, tumour pathogenicity, immunotherapy, biomarkers, targeted therapy

## Abstract

**Simple Summary:**

Despite significant advances in immunotherapy seen in the last decade, melanoma accounts for 2500 deaths a year in the UK. There remains an unmet clinical need to improve melanoma treatment. Melanoma is known as the “archetypal immunogenic tumour”, with a dense immune infiltrate. Chemokines are chemoattractant cytokines, essential for the positioning of all immune cells. This review outlines how the interplay of chemokine networks can enable melanoma tumours to survive, grow, metastasise, and evade anticancer immune responses. By better understanding how melanomas can exploit chemokine pathways, new targets to therapy may be revealed.

**Abstract:**

The incidence of cutaneous malignant melanoma is rising globally and is projected to continue to rise. Advances in immunotherapy over the last decade have demonstrated that manipulation of the immune cell compartment of tumours is a valuable weapon in the arsenal against cancer; however, limitations to treatment still exist. Cutaneous melanoma lesions feature a dense cell infiltrate, coordinated by chemokines, which control the positioning of all immune cells. Melanomas are able to use chemokine pathways to preferentially recruit cells, which aid their growth, survival, invasion and metastasis, and which enhance their ability to evade anticancer immune responses. Aside from this, chemokine signalling can directly influence angiogenesis, invasion, lymph node, and distal metastases, including epithelial to mesenchymal transition-like processes and transendothelial migration. Understanding the interplay of chemokines, cancer cells, and immune cells may uncover future avenues for melanoma therapy, namely: identifying biomarkers for patient stratification, augmenting the effect of current and emerging therapies, and designing specific treatments to target chemokine pathways, with the aim to reduce melanoma pathogenicity, metastatic potential, and enhance immune cell-mediated cancer killing. The chemokine network may provide selective and specific targets that, if included in current therapeutic regimens, harbour potential to improve outcomes for patients.

## 1. Introduction: Melanoma and Associated Chemokine Networks

Melanoma treatment has been revolutionised in the last decade, with the introduction of small molecule inhibitors, targeting the MAPK pathway, and immunotherapy, in the form of checkpoint inhibition. The success of checkpoint inhibition, whereby monoclonal antibodies targeting cytotoxic T-lymphocyte-associated protein 4 (CTLA-4) and the programmed cell death-1 axis (PD-1/PD-L1), has demonstrated that the manipulation of the immune system can provide a valuable therapeutic avenue for treating immunogenic tumours such as melanoma [1,2]. Despite doubling the 5-year survival rate of advanced melanoma to ~50%, melanoma remains the most aggressive form of skin cancer: 2500 patients die from melanoma every year in the UK, with the incidence increasing, and projected to continue increasing, for the next 15 years [3].

Melanomas arise from melanocytes that have acquired multiple pathogenic mutations bestowing upon the tumour the ability to proliferate, grow, resist apoptosis, and invade local structures, as well as eventually metastasise [4]. Underpinning these hallmarks of cancer is the establishment of a permissive tumour microenvironment (TME), a site akin to aberrant chronic inflammation, with a dense infiltrate of immune cells. Chemokines control the migration and location of all immune cells [5]. Their function is not only necessary for the recruitment of immune cells in inflammation and anti-tumour immune surveillance but also essential for immune cell development and tissue homeostasis [5]. Different subsets of immune cells express distinct chemokine receptors, thus allowing them to respond to precise combinations of chemokines secreted in specific tissues in response to particular infectious, inflammatory, or injury signals [5]. These mechanisms of cell migration are essential to drive effective antitumour immunity. However, chemokine pathways can be co-opted by tumour cells to drive “protumour effects”, such as the recruitment of immune suppressor cells and remodelling of the TME, and the promotion of tumour growth and metastasis [6].

Currently, a significant proportion of patients with melanoma fail to respond to checkpoint inhibition; in addition, 50% of treated patients experience adverse effects [7]. In particular, toxicities associated with immune activation and autoimmunity require medical treatment [8], with some long-term side effects (principally endocrinopathies) likely to be underrepresented in clinical trials [9]. The chemokine network represents a promising pathway for therapeutic interventions tailored to both enhancing antitumour immunity by promoting the recruitment and infiltration of tumour tissue by effector cells and blocking protumour functions by restricting the recruitment of immunosuppressive cells. Understanding and eventually manipulating the key axes controlling these processes may be of significant therapeutic relevance, particularly in the treatment of the archetypal immunogenic tumour melanoma by enabling immune cell-targeted interventions to be refined, thus creating treatments that carry fewer adverse effects and greater clinical efficacy.

In this review, we discuss the current knowledge of how cutaneous melanoma can exploit chemokine axes, and we will explore the latest and potential future roles for chemokine networks in melanoma therapy.

## 2. Tumour Establishment and Recruitment of Innate Immune Cells Which Aid Melanoma Growth

**Figure 1:**
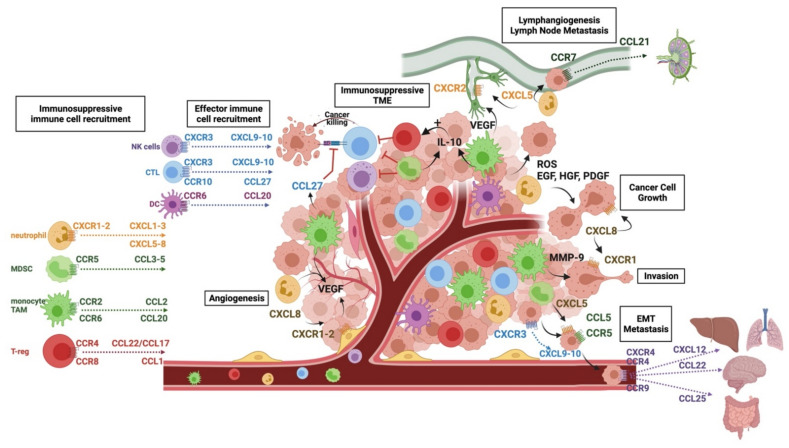
Chemokine networks in cutaneous melanoma. Immune cells are recruited via the networks outlined on the left of the figure. Chemokines enable an immunosuppressive microenvironment (TME) to be created via the preferential recruitment of immunosuppressive cells such as MDSCs, TAMs, and T-regs, which reduce the T-cell effector function and enhance the further recruitment and expansion of immunosuppressive cells. Cancer growth, invasion, and angiogenesis are promoted by the factors secreted by cells recruited to the TME via chemokines such as neutrophils and TAMs, as well as directly via CXCL8; lymphangiogenesis and lymph node metastasis are enabled through CXCL5/CXCR1-2 signalling, and cancer cells are able to migrate to lymph nodes using the CCL21/CCR7 pathway. EMT-like processes are enabled through CCL5 and CXCL5 signalling through their respective receptors. Transendothelial migration is promoted via the CXCL9-10/CXCR3 pathways, and cancer cells are able to move to specific sites using chemokine networks, e.g., CCL25/CCR9 to the gut, CCL22/CCR4 to the brain, and CXCL12 CXCR4 to the liver and lungs.

The ability of melanomas to grow and locally invade is key to their pathogenicity, demonstrated by the current staging system taking into account tumour thickness and local invasion [10,11]. The establishment and growth of melanoma tumours rely on a permissive TME able to recruit immune cells that support melanoma growth through the factors they secrete. Many chemokines are involved in the process, as outlined in Table 1 and graphically in Figure 1. Most of our knowledge of chemokine networks in melanoma come from animal models, whereby chemokines are blocked or genetically knocked out, or from immunohistochemical analyses of patient tissue samples throughout disease development and treatment.

### 2.1. CCR2 and Tumour Establishment

Chemokines demonstrate their importance in tumour growth from the very first steps of tumour initiation. Most melanomas are caused by UVB radiation inducing DNA damage and mutagenesis [4]; however, UVB is also able to activate melanocytes to upregulate their expression of ligands of chemokine receptor CCR2 [12]. In a study using neonatal mice exposed to UVB, Zaidi et al. [12] demonstrated that melanocytes exposed to UVB upregulated the expression of chemokines, including CCL2 and CCL7. This, in turn, recruited CCR2^+^ immune cells, most notably macrophages and myeloid-derived suppressor cells (MDSCs), which are able to support melanoma growth through multiple mechanisms.

Macrophages are a highly diverse and plastic class of tissue-resident phagocytes that are derived either during embryonic development from foetal liver or bone marrow precursors or following monocyte recruitment to adult tissues [13]. Monocyte-derived macrophages express multiple chemokine receptors, including CCR2, allowing their recruitment into the TME [14,15,16,17,18]. Tumour-associated macrophages (TAMs) can be polarised by the melanoma TME towards a subset that aids melanoma growth. TAMs secrete growth factors, such as macrophage inhibitory factor (MIF), which is able to retard cell cycle progression, as well as inhibit stress-induced apoptosis [19], and immunosuppressive cytokines, such as IL-10 [16]. TAMs can also contribute to feedback loops that further the recruitment of protumour innate cells: CCR6 is expressed by monocytes and dendritic cells (DCs) and is an important chemokine receptor mediating their infiltration into inflamed tissues, where CCL20 is produced in response to inflammatory stimuli [5]. Melanoma cells, which upregulate CCR6, are able to establish a paracrine loop between themselves and CCL20-expressing TAMs, promoting tumour growth and survival, likely in part due to TAMs promoting protumour immune cell infiltration [20]. CCL20^+^ TAMs can also secrete tumour necrosis factor-alpha (TNFα) and vascular endothelial growth factor-A (VEGF-A), contributing to tumour growth and angiogenesis. The presence of CCL20^+^ TAMS in melanoma tumours is associated with a poorer prognosis and reduced patient survival [21,22].

### 2.2. CXCL8 and Tumour Establishment

As well as the ligands of CCR2 being important in melanomagenesis, UV radiation has been shown to increase the secretion of CXCL8 (formerly known as IL-8), perhaps one of the most important chemokines for melanoma growth and survival [23]. CXCL8 is a ligand of the chemokine receptors CXCR1 and CXCR2. Immunohistochemical analyses of patient samples show an increased expression of CXCL8 and its receptors as melanomas transition from a radial (early stage) to vertical (later stage) growth phase [24], with CXCR1 upregulation being a key genetic difference between benign naevi and malignant melanoma [25]. CXCR1 and CXCR2-overexpressing tumours show an increased proliferation of melanoma cells and increased microvessel density, with evidence of reduced apoptosis in both cell lines in in vitro and in vivo models [26,27,28,29]. One mechanism underlying this is the ability of CXCL8 to promote melanoma cell survival via activating the phosphoinositide 3-kinase (PI3K)/Akt and mitogen-activated protein kinase (MAPK) signalling pathways, key signalling pathways involved in tumour cell survival and proliferation. This has been demonstrated using small molecule inhibitors of CXCR1/2, which are able to abrogate melanoma cell motility and to induce apoptosis by inhibiting the Akt pathway [30,31,32]. Aside from its direct effect on melanoma cells, CXCL8, along with other ligands of CXCR1 and CXCR2, namely CXCL1-3 and CXCL5-7, recruit innate cells, most notably neutrophils, which express the receptors CXCR1 and CXCR2 and are the “first responders” to tissue injury and damage [33]. Although there are few studies that have assessed the function of neutrophils within melanoma tumours, their presence is correlated with poorer prognosis and poor response to checkpoint inhibition [34,35]. They likely have a complex role in the TME, with both anti- and protumour functions. In other solid cancers, neutrophils support tumour growth by contributing to genetic instability by releasing reactive oxygen species (ROS) and secreting growth factors, such as epidermal growth factor (EGF), hepatocyte growth factor (HGF), and platelet-derived growth factor (PDGF) [36,37]. Tumour-associated neutrophils are also able to produce CXCL8, thus creating a feedback loop for further neutrophil recruitment. When this chemokine axis is inhibited in mouse models, melanoma infiltration by neutrophils is reduced, and reduced tumour growth, angiogenesis, and metastasis is seen, suggesting that neutrophils contribute to the protumour properties of CXCL8 and its receptors [26,27].

**Table 1:** Chemokines in melanoma: chemokines, their receptors, the cells on which they are expressed, and their pro- and antitumour functions in melanoma. Citations refer to preclinical studies that have determined the expression and role of chemokines and their receptors in a melanoma context.

Chemokine	Receptor	Ligand Expressed by	Receptor Expressed by	Protumour Effects	Antitumour Effects	Refs
CXCL1-3	CXCR1 CXCR2	Melanoma	Neutrophils MDSCs Transduced TILs	Tumour growth and survival Innate cell recruitment EMT Innate cell recruitment Lymphangiogenesis Lymph node metastasis	TILs recruitment (if transduced)	[23,24,26,27,31,35,38,39,40,41,42,43,44]
CXCL5-7						
CXCL8 (IL-8)				Tumour initiation Tumour growth and survival Innate cell recruitment Angiogenesis Invasiveness		
CXCL9-11	CXCR3	DCs Melanoma	T eff cells NK cells Melanoma	Transendothelial metastasis	TILs recruitment NK recruitment	[5,45,46,47,48]
CXCL12	CXCR4	Melanoma	T eff cells DCs Hepatocytes Melanoma	Metastasis—lung and liver		[49,50,51,52,53,54,55,56]
CCL2	CCR2	Melanoma	Monocytes TAMs MDSCs	Tumour initiation Tumour growth and survival Recruitment of innate cells		[12,16,18]
CCL3 CCL4 CCL7 CCL5	CCR1 CCR3 CCR5	Melanoma	MDSCs T cells	Immune evasion EMT		[57,58,59,60,61,62]
CCL17 CCL21	CCR4	Brain Melanoma	Melanoma T eff cells T regs	Immune evasion Metastasis—brain	TILs recruitment	[63,64,65,66,67,68]
CCL20	CCR6	TAMs	Melanoma TAMs MDSC	Innate cell recruitment		[20,21,22]
CCL19 CCL21	CCR7	HEVs LECs Melanoma	Melanoma T cells DCs	Lymph node metastasis	Antigen presentation TILs recruitment	[69,70,71,72,73,74,75,76,77]
CCL1	CCR8	Melanoma	T regs	Immune evasion		[63,64,65,66,67]
CCL25	CCR9	Small bowel	Melanoma	Metastasis—bowel		[78]
CCL27	CCR10	Melanoma	Melanoma T eff cells	Tumour cell survival Immune evasion	TILs recruitment	[79,80]
CX3CL1	CX3CR1	Melanoma	T eff cells	Angiogenesis	TILs recruitment	[81]

## 3. Chemokine Signals Enabling Immune Evasion

Once a melanoma tumour is established, its survival is dependent on its evasion of the antitumour immune response by recruiting melanoma-promoting, immunosuppressive immune cells and reducing the infiltration, activation, and proliferation of proinflammatory antitumour immune cells. The prognostic value placed not just on the degree of immune infiltration but the immune cell profile present in the TME reflects the importance of this selective recruitment. For example, a large scale study by Weiss et al. [82] demonstrated tumours with a “brisk” lymphocyte response where tumour-infiltrating lymphocytes (TILs) were present throughout the vertical growth phase, predicts better patient outcomes than “non-brisk” tumours, where TILs were present in fewer loci or “absent”. This classification has now been incorporated into the melanoma-reporting dataset. On the other hand, the presence of innate cells such as TAMs and neutrophils can predict poorer outcomes, as already mentioned, and indeed, the ratio of lymphocytes to innate cells is being explored as a marker of disease progression [83,84,85].

The main function of chemokine axes is to induce the recruitment and retention of immune cells within tissues, and melanoma cells are able to secrete selective chemokines that target immunosuppressive immune cells, including tumour-associated neutrophils, MDSCs, and regulatory T cells (T-regs), to preferentially recruit such cells over proinflammatory effector cells such as cytotoxic T-lymphocytes (CTLs).

### 3.1. Myeloid-Derived Suppressor Cells

CCR5 is a well-documented marker for Th1-type CD4+ T cells [86]. However, recent studies have also emphasised its role as a marker for immunosuppressive cells, including MDSCs, which produce inhibitory molecules, such as adenosine, nitric oxide, ROS, IL-10, transforming growth factor-beta (TGFβ), and PD-L1 [57]. In a series of studies in both mouse models and patients, Blattner et al. demonstrated the importance of CCR5 as a negative predictor of survival for melanoma [58]. CCR5^+^ MDSCs are found at high frequency in the melanoma TME, where the CCR5 ligands, CCL3-5, are also expressed. CCR5^+^ MDSCs are strong inhibitors of CD8^+^ CTLs [59], and their presence is associated with melanoma progression: patients with melanoma have a significantly increased frequency of circulating CCR5^+^ MDSCs compared with healthy volunteers [60]. Alongside, MDSCs and CCR5 ligands are expressed at higher frequencies in the TME when compared to the serum of the same patients [58]. Neutralising CCR5 in mouse models of melanoma leads to an increased survival time, reduced total numbers of MDSCs, and reduced immunosuppression by MDSCs, without diminishing the number of effector T cells infiltrating the tumour. A tumour-promoting function of CCR5 has also been established in preclinical models: CCR5 knockout mice have significantly smaller tumours than CCR5^+/+^ mice when inoculated with B16 melanoma cells, with an increased infiltration of CTLs and natural killer (NK) cells in tumours [61].

### 3.2. Regulatory T Cells

The recruitment and expansion of T-reg populations within the TME of melanoma is associated with a poor prognosis [63]. T-regs are able to contribute to the immunosuppressive environment by secreting cytokines, such as TGF-β, IL-10, and IL-35, and by consuming IL-2 and expressing immune checkpoint inhibitors, thereby decreasing effector T-cell functions [66]. Their expansion can be promoted by factors secreted by immune cells within the TME, such as IL-10 secreted by MDSCs and TAMs [13,60], or they can be recruited via chemokine pathways.

Both CCR4 and CCR8 are prominent chemokine receptors expressed by T-regs: CCR4 is broadly expressed on T-regs in healthy tissues throughout the body, whereas CCR8 is more selectively expressed by cutaneous T-regs and those present in the TME [64,87]. CCL1 and CCL22, the main ligands for CCR8 and CCR4, respectively, have been shown to be upregulated both at the gene and protein levels in the TME of melanoma, suggesting that melanoma cells are able to actively recruit T-regs [66,67]. The anti-CCR4 monoclonal antibody mogamulizumab can reduce the number of circulating T-regs in patients with solid tumours [88], although, due to its broad expression in T-regs and conventional T cells, treatment is associated with autoimmune toxicity [89]. A large study by Plitas et al. [64] demonstrated that CCR8 is upregulated on highly suppressive T-regs in the TME of breast cancer and melanoma and suggest that CCR8 could prove to be a useful target. Interestingly, CCR8 appears to be expressed on T-regs after TCR-mediated activation [90], and yet, it does not appear to be required for recruitment into melanoma tissues. Indeed, systemic CCR8 ablation had no effect on the infiltration and immunosuppressive function of T-regs in murine melanoma, contrary to previously held assumptions [91]. For more details, please consult the article written by B Moser for this Special Issue.

### 3.3. Creation of an Immunosuppressive TME

The manipulation of chemokine axes in melanoma can also be used to create a more immunoregulatory environment independent of immune cell recruitment. CCR10 has been implicated in directly providing melanoma with resistance to cytotoxic immune cells. This is in sharp contrast to its usual role as an important receptor for T-cell homing to cutaneous tissue, with its ligand, CCL27, produced by keratinocytes in the skin but also expressed by melanoma cells [79]. Simonetti et al. [80] observed that the expression of CCR10 and its ligand CCL27 are associated with thicker tumours and a reduced density of infiltrating lymphocytes, with CCR10 overexpression enhancing tumours’ potential to grow and evade host immune responses. When CCL27 was added, signalling through CCR10 led to activation of the Akt and PI3K pathways, enabling melanoma cells to resist Fas-mediated apoptosis, a key mechanism in tumour cell clearance. This mechanism may also lead to the internalisation of CCL27, perhaps thereby reducing the chemoattraction of effector T cells.

## 4. Angiogenesis

The oxygen and nutrients essential for tumour growth are only able to penetrate tissues about 100–200 μm deep from existing capillaries; therefore, a rich vascular network is essential for continued local tumour growth, especially in the vertical growth phase [92]. The most significant chemokine in angiogenesis in melanoma is CXCL8, which can have paracrine and autocrine effects when it binds to CXCR1 and CXCR2, expressed on melanoma cells and endothelial cells within the TME [24,31]. CXCL8 can promote the proliferation of endothelial cells, their ability to form capillary tube-like structures, and their migration. By signalling through CXCR1 and CXCR2 expressed on endothelial cells, CXCL8 is also able to upregulate VEGF expression, as well as upregulate the expression of metalloproteinases (MMPs). MMPs are able to degrade the extracellular matrix (ECM), which not only contributes to tissue remodelling and growth but leads to the release of ECM-bound growth factors, aiding further growth and angiogenesis [92]. Several studies have demonstrated that, in addition to CXCL8, the ligands CXCL1-3 can induce angiogenesis by involving the thrombin pathways [39,40]. When the CXCR1/CXCR2 signalling pathway is blocked, new vessel formation is greatly reduced, and melanoma growth is inhibited [26,28,40].

Angiogenesis can also be promoted by innate cells following their recruitment into the TME. Both TAMs and neutrophils can contribute to angiogenesis directly via the secretion of VEGF, the most potent angiogenic factor, and MIF [19,37,93]. TGFβ, also secreted by TAMs and abundant within the TME, can promote the upregulation of VEGF and the expression of CXCL8 [94], as can macrophage-derived TNFα and IL-1, further contributing to angiogenesis [95].

## 5. Invasion and Metastasis

Metastasis is the main cause of death from human cancer [96,97]. Metastasis is a process that requires several steps: the invasion of local adjacent tissue by tumour cells, migration into the lymphatic system, intravasation of tumour cells into blood vessels, survival and circulation through the blood, extravasation at distant sites, and finally, the initiation of secondary tumours in distant organs, which requires appropriate conditions for proliferation and neo-vascularisation, the so-called establishment of a metastatic niche [96].

### 5.1. Invasion

CXCL8 plays a key role in promoting melanoma motility [23], and when CXCL8 is blocked, melanoma invasiveness is greatly reduced [41]. CXCL8 signalling through CXCR1 stimulates phospholipase C signalling, which, in turn, leads to regulation of the actin cytoskeleton through the phosphorylation of protein kinase C; aside from this, CXCR1 signalling causes Rho-GTPase-induced polymerisation of actin cytoskeletons [31], both of which promote cell motility. Immune cells recruited to the TME via chemokine pathways can also promote invasion through the factors they secrete. For example, TAMs upregulate urokinase-type plasminogen activator (uPAR) and secrete MMPs, such as MMP-9, which enable the remodelling of the ECM [98] and increase the invasiveness of melanoma, or they upregulate such factors in melanoma cells through the secretion of TNFα and IL-1α [98].

### 5.2. Lymphangiogenesis and Lymphatic Metastasis

Lymph node metastasis represents one of the first key steps in the dissemination of melanoma to distant sites. Lymphangiogenesis, as measured by lymphatic vessel density (LVD), is associated with increased lymph node metastasis, with several studies supporting the hypothesis that new lymph vessel formation can be actively induced in melanoma and that this promotes metastasis to lymph nodes [99].

The mechanisms behind lymphangiogenesis in melanoma are complex and still to be precisely defined. However, it is clear that the excretion of VEGF isotypes VEGF-C and VEGF-D, and their binding to VEGFR-3 on lymphatic endothelial cells (LECs), is essential for the formation of new lymphatic vessels. Within the TME of melanoma, sources of VEGF include TAMs and neutrophils, recruited via the chemokine pathways outlined above [16,37].

Chemokines may also play a more direct role in the development of new lymph vessels: in immortalised human lymphatic endothelial cells, CXCL5 signalling through CXCR2 was able to induce lymphatic sprouts to a similar extent as VEGF-C [42]. When studying this effect further, Soler Cardona et al. [42] noted that CXCR2 is expressed by human lymphatic endothelial cells, and the expression of CXCL5 correlates with increased neutrophil density and an increased risk of locoregional metastasis. Neutrophils appear to aid tumour intravasation into lymphatic vessels, with a 3.5-fold increase in tumour cell migration when neutrophils were present in an in vitro model [42]. Although the exact mechanisms are yet to be elucidated, the presence of neutrophils in predicting the likelihood of locoregional metastases is being widely studied [84].

The CCR7/CCL21 pathway coordinates the migration of melanoma cells towards and into lymphatic vessels. It is well-established that immune cells use CCR7 to migrate into lymph nodes [100]. This mechanism applies to both circulating naïve and central memory T cells entering lymph nodes via high-endothelial venules (HEVs), as well as to tissue-derived memory T cells and DCs via the draining lymphatic system [5]. CCL19 and CCL21, the ligands for CCR7, are strategically expressed by LECs, HEVs, and stromal cells within lymph nodes [69,70]. The CCR7 axis enables the co-localisation of mature, antigen-presenting DCs, and T cells bearing cognate T-cell antigen receptors and, thus, provides a core mechanism for the initiation of cellular and humoral immunity in response to specific antigenic challenges [5,69]. Melanoma cells exploit this same chemokine axis by upregulating CCR7 expression, enabling migration towards CCL21-producing LECs [71]. The overexpression of CCR7 on melanoma cells caused a greater migration of these cells towards LECs, and the use of a CCL21-neutralising antibody stopped their migration in vitro. In vivo models have replicated these findings with CCR7-transduced B16 melanoma cells demonstrating greater metastasis to lymph nodes [72,73].

### 5.3. Epithelial to Mesenchymal Transition

Epithelial to mesenchymal transition (EMT) is a process by which tumour cells lose their tissue residency, cell polarity, and cell–cell junctions and upregulate mesenchymal markers, gaining a mesenchymal-like phenotype that enhances migration, invasiveness, and resistance to apoptosis. EMT leads to the disruption of the integrity of the basement membrane and the ability to tumour cells to migrate to other tissues [101]. Several transcription factors and signalling pathways have been implicated in controlling EMT, and chemokines have been demonstrated to influence these signalling pathways in a variety of solid tumours.

In melanoma, although it is not an epithelial tumour, an EMT-like process may still occur, referred to as “phenotype switching”. The signalling of CCL5 through CCR5 has been shown to positively regulate TGFβ, which, in turn, induces EMT through PI3k/AKT/GSK3b signalling [62], maintaining a mesenchymal phenotype and the metastatic properties of melanoma cells. CXCL5 has been shown to contribute to EMT in several solid tumours, including gastric, colorectal, and hepatocellular, by enhancing the signalling pathways required by EMT [102,103] but, also, by recruiting and activating neutrophils, which are then able to enhance the invasive properties of cancer cells via the factors they secrete [104]. Similarly, in melanoma, MDSCs recruited via CXCL5 have been shown to induce EMT by producing factors such as TGF-β, EGF, and HGF, and when depleted, the rate of metastasis in animal models is greatly reduced [43].

### 5.4. Transendothelial Metastasis

Chemokine signalling is also involved in intravasation, a process whereby mobilised tumour cells directly access blood via reverse transendothelial migration [105]. The work by Amatschek et al. [47] on excised melanoma metastases demonstrated that tumour microvessel endothelial cells express high levels of CXCL9 and CXCL10 and confirmed that melanoma cells express CXCR3. CXCR3 is important for the migration of NK cells and has an important role in CD4^+^ Th1 cell priming and the recruitment of CD4^+^ and CD8^+^ effector T cells [5]. Using a series of in vitro migration assays, Amatschek et al. showed that melanoma cells can migrate towards the CXCL9 gradients created by tumour endothelial cells and through endothelial monolayers, a process accelerated by additional soluble CXCL9. Upon stimulation with CXCL9, melanoma transmigration led to the formation of “holes” in the endothelium, caused by the disruption of cell–cell contact. This process was blunted by anti-CXCL9 and anti-CXCR3 antibodies, suggesting that the CXCR3-CXCL9/10 axis plays a crucial role in this step of cancer dissemination, with further studies demonstrating that CXCL10/CXCR3 co-expression is associated with early metastatic disease progression and poor overall survival [45,48].

Once in the blood, melanoma cells again depend on chemokines for their recruitment to specific organs. The location of secondary tumour growth depends upon the chemokine receptors expressed on tumour cells and the chemokine expression that is specific to certain tissue sites. For example, the CXCR4–CXCL12 axis has been associated with pulmonary, as well as liver and bone marrow, metastases [49,55]. The expression of CXCR4 by melanoma cells in patient samples has been correlated with the likelihood of pulmonary metastasis [50,51] and increased pulmonary metastasis has been seen in mice inoculated with melanoma cells overexpressing CXCR4 [52,53,54], with CXCR4 inhibition reversing this effect [56]. The binding of chemokines to their receptors induces inside-out signalling, leading to affinity changes in integrins, which is a prerequisite for cell attachment and subsequent transendothelial migration [106]. This mechanism may explain the recruitment of CXCR4-expressing tumour cells in response to CXCL12 displayed on the inner walls of the microvessels via the engagement of β-integrins with VCAM-1 (vascular cell adhesion molecule 1) expressed on endothelial cells [107]. In mouse models, the blockade of CXCR4 or CXCL12 in the early course of metastasis reduced the number of pulmonary metastases; however, if the blockade happened later, the sizes of the metastases were reduced. These findings suggest that CXCR4 may also play a role in the early stages of the establishment and growth of metastases.

As CXCR4 is thought to promote migration to the lungs, liver, and bone marrow, other chemokines are associated with metastasis to specific organs: functionally active CCR9 facilitates metastasis of mouse tumour cells to the small bowel [78], and CCR4 appears to play an important role in metastasis to the brain [68], the site at which CCL22, one of its ligands, is expressed.

## 6. How Can Chemokines Be Exploited Therapeutically?

Chemokines could prove to be useful tools in the therapeutic arsenal against melanoma. Specifically, by: (1) aiding in the stratification of treatment and “personalised medicine”, (2) improving the efficacy of current cellular therapeutics, and (3) directly targeting specific subsets of cells or tumour-promoting migratory pathways. Monoclonal antibodies and small molecule inhibitors have been developed to block the interactions between chemokines and their receptors. For example, the anti-CCR4 monoclonal antibody mogamulizumab is already clinically approved to treat haematological malignancies [88] and may also be applied to the treatment of melanoma. On the other hand, where appropriate, an increased chemokine expression may help achieve antitumour immune activation by local administration, for example, as an adjuvant to DNA vaccines [75], and where chemokine receptor expression is desirable, chemokine receptors can be transduced in cells used for autologous transfer [44].

### 6.1. Diagnosis, Disease Stratification, and Monitoring

As this review clearly outlines, melanoma cells differ from healthy tissue in the chemokines they express. Immunohistochemistry and genetic studies, as well as analyses of patient serum, have highlighted these differences and could be incorporated to aid diagnosis. For example, malignant melanomas express higher levels of CXCL1, CXCL2, and CXCL8 and receptors CXCR1, CXCR4, CCR10, and CCR7 compared with benign naevi [108,109,110,111]. The expression of chemokines such as CXCL8 and its receptor CXCR2 increase as the tumour transitions from the radial to the vertical growth phase [24]. Chemokine expression also correlates with prognosis; the expression of CXCR3 by melanoma cells is associated with a poorer prognosis and increased chance of metastasis [48], whereas CCL27 expression in the supra-tumoral dermis is associated with longer progression-free survival, perhaps due to the recruitment of CCR10^+^ lymphocytes [112]. The contradictory results to Simonetti et al.’s work demonstrate the complex nature of chemokine pathways; CCR10 expression by melanoma cells appears to be associated with a poorer prognosis due to signalling through CCR10 enabling the evasion of apoptosis [113], whereas the expression of its ligand CCL27 likely promotes the recruitment of CCR10^+^ effector T cells [112], underscoring the context dependence of chemokine functions.

As well as this, chemokine expression can be used to monitor responses to treatment. Increasingly high dimensional techniques, such as CyTOF and single-cell RNA-Seq, allow multiple chemokines to be analysed in a large number of cells from individual patients. Some groups have already conducted such studies using publicly available datasets of the transcriptomes of skin cancers [114,115], and chemokine profiles are being included as secondary outcomes in clinical trials to identify biomarkers of responses to treatment (see Table 2). Evidence is emerging for a role for chemokines as prognostic and predictive biomarkers of therapy responses; CXCL5 has been postulated as a biomarker for the response to anti-PD-1 therapy, with high baseline levels associated with the response to treatment [116], and serum CXCL8 has been shown to decrease in patients who develop good responses to immunotherapy [117]. CXCL8 has been shown in many studies to correlate with tumour burden; the levels of CXCL8 in the serum of tumour-bearing mice and humans fall following surgical excision [118], patients with brain metastasis express high levels of CXCL8 in cerebrospinal fluid [119,120], and CXCL8 has been shown to be higher in models of BRAF inhibitor (BRAFi)-resistant melanoma [121].

Changes in chemokine expression have been studied in mouse models of BRAFi-treated melanoma. When melanomas are sensitive to BRAFi treatment, CCL2 expression and production is reduced, which correlates with a reduction in MDSC infiltration [122]. Similar studies have shown that increases in CXCL9 and CXCL10 correlate with an enhanced infiltration of T cells, which decreases as tumours become resistant to BRAFi treatment [123].

### 6.2. Improving the Efficacy of Current Treatments of Melanoma

Chemokines could be used to improve current therapies for melanoma, and as mentioned, there remains a need for alternatives or adjuvants to current treatments. Adoptive cell therapy, (ACT), and, specifically, chimeric antigen receptor (CAR-) T-cell therapy, whereby autologous T cells are genetically transduced with a tumour-specific CAR, expanded and reinfused in the patient, has shown great success in haematological malignancies but is still being developed in solid tumours such as melanoma [124]. The transduction of chemokine receptors may overcome the current issues of ACT, namely by increasing the T-cell infiltration of tumours whilst maintaining antigen selectivity, thus reducing the burden of autoimmune side effects. CXCR1 and CXCR2 have been shown to improve the infiltration of CTLs [44,125], and CXCR2-transduced T cells are being trialled in a clinical pilot study [NCT01740557]. CXCR3 is not only essential for T-cell trafficking to melanoma but can improve NK-cell infiltration of CXCL10-producing tumours [126]. Additionally, the ex vivo expansion of NK cells can itself increase NK expression of CXCR3, which is further enhanced by exogenous IFN-α [127]; thus, the process of generating NK cells for ACT increases their ability to infiltrate tumours. Chemokine modulation may also improve DC-based ACT and is currently being explored in a phase 2 clinical trial (NCT04093323) (see Table 2).

Further to this, direct chemokine administration could also be a useful adjunct to current therapies. CXCL10 secreted by DCs can recruit effector T cells into the TME [46]. CXCL10 is also secreted at higher levels by PBMCs in patients in remission [128]. When CXCL10 is administered via an adenovirus vector in mouse models of melanoma, reduced tumour growth is seen [128]. These data raise the possibility that CXCL10 could increase the frequency of CXCR3^+^ TILs and could act as an adjunct to current treatments, including ACT and checkpoint inhibition.

We have already discussed how melanoma is able to exploit the CCR7/CCL21 axis, but efforts have also been made to use this axis for therapeutic benefit. CCR7 is used by immune cells to enter lymph nodes, the site of antigen presentation and initiation of T-cell responses [70]. An enhanced cytotoxic activity of adoptively transferred T cells is seen when T cells are expanded ex vivo in the presence of CCL21. This has been shown to result in improved infiltration of these T cells into tumours and in increased tumour suppression in in vivo models [77]. In addition to this work, several groups have demonstrated that CCL21 administration increases the frequency of TILs: Yamano et al. [75] used CCL21 as an adjuvant to promote the effect of a melanoma DNA vaccine, and Chen et al. [76] used an adenovirus vector expressing CCL21 in combination with paclitaxel and demonstrated that the combination was better than either treatment alone. Using CCL21 as an adjunct was associated with increased tumour infiltration by immune cells, improved tumour cell apoptosis, and reduced blood vessel formation in the tumour tissue, but this is yet to be repeated in combination with more recent treatments, such as checkpoint inhibition or MAPK inhibitors.

Recruiting cytotoxic immune cells is one strategy to improve current therapies; however, preventing the recruitment of immunosuppressive cells could be another. A CXCR1/CXCR2 small molecule inhibitor is currently in a phase 1 clinical trial in combination with checkpoint inhibition (NCT03161431) to reduce innate immune cell recruitment. In preclinical trials, anti-CCL2 in combination with BRAF-targeted therapy reduced tumour sizes [122], as did anti-CXCR4 peptides when used alongside PD-1 [129], and both demonstrated a reduction in MDSCs and T-regs. Furthermore, based on the key roles of CCR5 on both melanoma cells and MDSCs, blocking CCR5 may help to: (a) limit the metastatic potential of melanoma cells, (b) reduce the number of immunosuppressive cells within the TME, and (c) enhance the infiltration of CTLs into tumours. Although these pathways have not yet been successfully targeted in clinical trials in patients with cancer, and there is a paucity of clinical trials targeting chemokine pathways in patients with melanoma (see Table 2), there are clinical trials targeting chemokines for many other cancer types in cancers where checkpoint inhibition alone has failed to achieve the revolution seen in melanoma therapy. The blockade of CCR5 is being extensively explored in colorectal cancer (NCT04721301) and triple-negative breast cancer (NCT03838367), CCR2 blockade is being explored in pancreatic (NCT03767582) and lung cancer (NCT04123379), and CXCR4 antagonists in haematological malignancies (NCT01740557).

### 6.3. Direct Targeting of Specific Migratory Pathways

Targeting chemokines could be a therapeutic approach in its own right. As mentioned above, several treatment approaches, including small molecules, peptides, or antibodies against CXCR4, have shown great promise at reducing the burden of metastasis in preclinical studies [129]. In a similar vein, Emmett et al. showed that blocking CCR7 can reduce melanoma migration towards and infiltration of lymph nodes [73]. This appears to be in contrast with the above idea of using CCL21 as an adjunct to promote the recruitment of CTLs, which also express CCR7. Indeed, in a mouse model of melanoma, Wetzel et al. [130] delivered CCR7 via a parvovirus, which led to the increased activation of T cells and NK cells and tumour shrinkage. This is a clear example of how melanomas can be targeted therapeutically through the same chemokine pathways that are known to increase melanoma pathogenicity and cancer metastasis. In a therapeutic setting, manipulating these chemokine networks can be finetuned to instead activate immune responses and enhance cancer killing. For instance, when melanoma cells express the receptor CCR7, they are able to migrate along the same ligand gradients as circulating immune cells to enter lymph nodes; however, when CCL21 is expressed by melanoma, immune cell recruitment to the TME via CCL21 gradients is magnified. The manipulation of such axes therefore requires great care in order to prevent the disruption of tissue homeostasis and preserve the important functions of such networks.

CCR4 is a second target with potential dual effects; CCR4 contributes to melanoma metastasis to the brain [68], and it is also expressed on the most suppressive tumour-infiltrating T-regs [66,131]. As already mentioned, the anti-CCR4 antibody mogamulizumab has been shown to deplete these highly suppressive T-regs and is used to target malignant T cells in adult T-cell lymphoma. CCR4, however, is not selective for T-regs and is also expressed by Th17 and Th2 cells, thought to be important in enhancing the antitumour CTL response [132]. Although anti-CCR4 treatment has the potential to reduce T-regs and their immunosuppressive effects, CCR4 blockade also has the potential to impair effector T-cell responses by reducing the Th17 induction of CTLs. The lack of specificity and redundancy in the chemokine network is highlighted by this potential target and reiterates the importance of striving to gain a thorough understanding of the distinct cellular responses controlled by chemokines.

As mentioned, CCR8 is thought to be a more selective marker for tumour-infiltrating T-regs, but the blockade of CCR8 does not appear to impact the number of T-regs infiltrating melanoma. Despite this, there is currently an anti-CCR8 monoclonal antibody in phase 1 clinical trials in solid tumours refractory to other treatments (NCT05007782).

## 7. Discussion: Challenges in Studying of Chemokine Networks and Implications for Clinical Translation

Although this review covers a significant body of work and some excellently crafted studies, it is important to iterate that most of these studies have been conducted in mouse models, some using mouse melanoma cell lines and others using human melanoma cell line xenografts in rodent models. It is well-established that murine and human chemokine networks differ and that murine and human chemokines do not always interact with each other [133]. This is particularly important in experiments where the interaction between a murine chemokine and human receptor or vice versa is being explored. Added to this, the anatomical structures of human and mouse skins differ, and indeed, laboratory mice in clean conditions have different resident immune cells in the skin when compared to humans [65,122]. How these cells contribute to the reaction towards a skin malignancy may not be fully replicated by solely interrogating such animal models.

As discussed above, it is important to recognise that a multitude of cells with different functions can express the same chemokines or receptors. Therefore, blocking a receptor may have unforeseen consequences. In many studies where the blockade of a chemokine axis has demonstrated an effect, transfected cells that overexpress the chemokine in question have been used. Although this technique is useful to help better explore the functions of certain chemokines, these experiments do not adequately recapitulate the patient setting, and the effect of the blockade can often be exaggerated. As we have discussed, the chemokine network is highly complex, and even if the blockade of overexpressed chemokines has an effect, this may not fully translate to the clinical setting.

Another challenge comes from chemokines not being selective for tumour-associated receptors. For example, targeting chemokines contributing to tumour-associated angiogenesis could also have an impact on normal vasculature and the blood vessel formation seen in wound healing [40,134,135], potentially causing toxicities. Using chemokines to block the recruitment of cells such as neutrophils and monocytes, which contribute to tumour growth, could also have an impact on the recruitment of such cells in inflammation or affect immune surveillance, and enhancing antitumour effector T-cell functions or blocking immunosuppressive T-reg functions could have unwanted autoimmune consequences, as seen with the adverse side effects of mogamulizumab treatment; for a more detailed discussion of CCR4-targeted treatments, please consult the article by O. Yoshie in this Special Issue. Autoimmunity is already an issue in immunotherapy, and although one of the ideas we have touched on is how targeting chemokines may make immunotherapy more selective, care is needed when manipulating such important signalling networks. Understanding the functions of chemokines in tissue homeostasis will not only help prevent adverse effects of chemokine manipulation but may also shed light on new ways in which the normal function of chemokines could be used for therapeutic gain.

All of these factors have most likely contributed to the lack of clinical translation of chemokines and their receptors as targets for cancer therapy. However, there are other issues that may be more specific to melanoma. For example, an area that is underexplored is how the chemokine profile of melanoma changes temporally as the disease progresses. Melanoma metastases preferentially target the same sites: brain, liver, and skin. The role of chemokines in metastasis is undeniable, but little is known about how the expression of chemokines and their receptors are regulated as melanomas gain metastatic potential. Secondly, cancer stem cells, a subset of self-renewing cancer cells that are able to initiate tumour growth, may play an important role in resistance to therapy [136]. Although under-researched, the role of chemokines in the maintenance and positioning of these cells may unearth new targets for inhibiting the tumorigenic functions of these cells [137]. In addition, melanoma is the most immunogenic tumour and appears to be reliant on the recruitment of immune cells for its progression, and yet, it appears to be a cancer with the fewest clinical studies of chemokine manipulation. This may be due, in part, to the success of checkpoint inhibitor therapies; most clinical trials in melanoma are aimed at enhancing these successes of checkpoint inhibition, whereas, in malignancies that have not benefitted from checkpoint inhibitors, new therapeutic avenues appear to be being explored more widely. Perhaps, it may be beneficial to revisit chemokine targets that were neglected as a consequence of the substantial progress achieved with checkpoint inhibitors.

## 8. Conclusions

The interplay of chemokine axes is essential for the growth, survival, invasion, and metastasis of melanoma. The successful manipulation of the key chemokine pathways alone or as part of treatment combinations by clinicians could provide novel tools to overcome this disease.

## Figures and Tables

**Table 2:** Current clinical trials targeting and monitoring chemokines in melanoma.

Targeting Chemokines						
Target	Agent name	Type of agent	Melanoma Type	Phase	Aims/Outcomes	Trial Number
CXCR1/CXCR2	SX-682	Small molecule inhibitor	Stages 3 or 4	1	Blocking recruitment of MDSCs	NCT03161431
CXCR2	Autologous TILs	CXCR2-transduced autologous TILs	Stages 3 or 4	Pilot	Increased recruitment of TILs	NCT01740557
Chemokine modulation	DCs, celecoxib, IFNa2b, rintatolimod	Autologous DCs + chemokine modulation	PD1/PD-L1-resistant	2	Increased immune response	NCT04093323
**Monitoring chemokines/chemokines as biomarkers**						
TLR	CMP-001 + pembrolizumab	TLR9 agonist + anti-PD-1 antibody	Recent or current PD1/PD-L1 therapy	1b	Activate DCs to augment immunotherapy Measuring CXCL10 as biomarker for response to treatment	NCT02680184
BRAF	Dabrafenib + trametinib	Neoadjuvant BRAFi	Stage 3 BRAF V600 mutant	2	Improve pathological response Measuring chemokines in tumour and peripheral blood to identify biomarkers for response to treatment	NCT01972347
Immune checkpoint	Immune checkpoint inhibitors	Immune checkpoint inhibitors	Stages 3 or 4 Current immune therapy	N/A	Measuring chemokines and immune profile in tumour and peripheral blood to identify biomarkers for response to treatment	NCT04576429
TILs	TBX-3400	AT-MYC fusion protein	Stages 3 or 4 Immune therapy-resistant	1	Activate cytotoxic T cells Measuring chemokines in peripheral blood to identify biomarkers for drug activity	NCT03385486
PD-1		Anti-PD-1 antibody	All stages Treatment naive	2	Measuring chemokine profiles to identify biomarkers for response to treatment	NCT04928365
TILs	IL-2	IL-2	Any stage Pretreatment	3	Increase TILs recruitment Assess immune cell infiltration and systemic immune response to IL-2 therapy, including measuring peripheral chemokines	NCT03233828
BRAF + immune checkpoint	Cobimetinib + vemurafenib + atezolizumab	BRAFi + anti-PD-L1-antibody + surgery	Stages 3 or 4 Resectable BRAF V600 mutant	2	Measuring chemokines and immune profile in tumour and peripheral blood to identify biomarkers for response to treatment	NCT04722575

## References

[1] Rodríguez-Cerdeira C., Carnero Gregorio M., López-Barcenas A., Sánchez-Blanco E., Sánchez-Blanco B., Fabbrocini G., Bardhi B., Sinani A., Guzman R.A. (2017). Advances in Immunotherapy for Melanoma: A Comprehensive Review. Mediat. Inflamm..

[2] Farkona S., Diamandis E.P., Blasutig I.M. (2016). Cancer immunotherapy: The beginning of the end of cancer?. BMC Med..

[3] UK C.R. Melanoma Skin Cancer Statistics, Cancer Research UK. https://www.cancerresearchuk.org/health-professional/cancer-statistics/statistics-by-cancer-type/melanoma-skin-cancer.

[4] Shain A.H., Bastian B.C. (2016). From melanocytes to melanomas. Nat. Rev. Cancer.

[5] Griffith J.W., Sokol C.L., Luster A.D. (2014). Chemokines and chemokine receptors: Positioning cells for host defense and immunity. Annu. Rev. Immunol..

[6] Jacquelot N., Duong C.P.M., Belz G.T., Zitvogel L. (2018). Targeting Chemokines and Chemokine Receptors in Melanoma and Other Cancers. Front. Immunol..

[7] Moreira A., Heinzerling L., Bhardwaj N., Friedlander P. (2021). Current Melanoma Treatments: Where Do We Stand?. Cancers.

[8] Wolchok J.D., Chiarion-Sileni V., Gonzalez R., Rutkowski P., Grob J.J., Cowey C.L., Lao C.D., Wagstaff J., Schadendorf D., Ferrucci P.F. (2017). Overall Survival with Combined Nivolumab and Ipilimumab in Advanced Melanoma. New Engl. J. Med..

[9] CH J., Warshauer JTBluestone J. (2017). Is autoimmunity the Achilles’ heel of cancer immunotherapy?. Nat. Med..

[10] Gershenwald J.E., Scolyer R.A., Hess K.R., Sondak V.K., Long G.V., Ross M.I., Lazar A.J., Faries M.B., Kirkwood J.M., McArthur G.A. (2017). Melanoma staging: Evidence-based changes in the American Joint Committee on Cancer eighth edition cancer staging manual. CA Cancer J. Clin..

[11] Roncati L., Piscioli F. (2018). AJCC 8th Edition (2017) versus AJCC 7th Edition (2010) in thin melanoma staging. Neoplasma.

[12] Zaidi M.R., Davis S., Noonan F.P., Graff-Cherry C., Hawley T.S., Walker R.L., Feigenbaum L., Fuchs E., Lyakh L., Young H.A. (2011). Interferon-γ links ultraviolet radiation to melanomagenesis in mice. Nature.

[13] Zhou J., Tang Z., Gao S., Li C., Feng Y., Zhou X. (2020). Tumor-Associated Macrophages: Recent Insights and Therapies. Front. Oncol..

[14] Coussens L.M., Werb Z. (2002). Inflammation and cancer. Nature.

[15] Ilkovitch D., Lopez D.M. (2008). Immune modulation by melanoma-derived factors. Exp. Dermatol..

[16] Pieniazek M., Matkowski R., Donizy P. (2018). Macrophages in skin melanoma-the key element in melanomagenesis. Oncol. Lett..

[17] Georgouli M., Herraiz C., Crosas-Molist E., Fanshawe B., Maiques O., Perdrix A., Pandya P., Rodriguez-Hernandez I., Ilieva K.M., Cantelli G. (2019). Regional Activation of Myosin II in Cancer Cells Drives Tumor Progression via a Secretory Cross-Talk with the Immune Microenvironment. Cell.

[18] Nesbit M., Schaider H., Miller T.H., Herlyn M. (2001). Low-level monocyte chemoattractant protein-1 stimulation of monocytes leads to tumor formation in nontumorigenic melanoma cells. J. Immunol..

[19] Soumoy L., Kindt N., Ghanem G., Saussez S., Journe F. (2019). Role of Macrophage Migration Inhibitory Factor (MIF) in Melanoma. Cancers (Basel).

[20] Kadomoto S., Izumi K., Mizokami A. (2020). The CCL20-CCR6 Axis in Cancer Progression. Int. J. Mol. Sci..

[21] Martin-Garcia D., Silva-Vilches C., Will R., Enk A.H., Lonsdorf A.S. (2020). Tumor-derived CCL20 affects B16 melanoma growth in mice. J. Dermatol. Sci..

[22] Samaniego R., Gutiérrez-González A., Gutiérrez-Seijo A., Sánchez-Gregorio S., García-Giménez J., Mercader E., Márquez-Rodas I., Avilés J.A., Relloso M., Sánchez-Mateos P. (2018). CCL20 Expression by Tumor-Associated Macrophages Predicts Progression of Human Primary Cutaneous Melanoma. Cancer Immunol. Res..

[23] Gebhardt C., Averbeck M., Viertel A., Kauer F., Saalbach A., Anderegg U., Simon J.C. (2007). Ultraviolet-B irradiation enhances melanoma cell motility via induction of autocrine interleukin 8 secretion. Exp. Dermatol..

[24] Varney M.L., Johansson S.L., Singh R.K. (2006). Distinct expression of CXCL8 and its receptors CXCR1 and CXCR2 and their association with vessel density and aggressiveness in malignant melanoma. Am. J. Clin. Pathol..

[25] Su W., Guan Y., Huang B., Wang J., Wei Y., Zhao Y., Jiao Q., Ji J., Yu D., Xu L. (2020). Bioinformatic analysis reveals hub genes and pathways that promote melanoma metastasis. BMC Cancer.

[26] Singh S., Varney M., Singh R.K. (2009). Host CXCR2-dependent regulation of melanoma growth, angiogenesis, and experimental lung metastasis. Cancer Res..

[27] Singh S., Nannuru K.C., Sadanandam A., Varney M.L., Singh R.K. (2009). CXCR1 and CXCR2 enhances human melanoma tumourigenesis, growth and invasion. Br. J. Cancer.

[28] Singh S., Sadanandam A., Nannuru K.C., Varney M.L., Mayer-Ezell R., Bond R., Singh R.K. (2009). Small-molecule antagonists for CXCR2 and CXCR1 inhibit human melanoma growth by decreasing tumor cell proliferation, survival, and angiogenesis. Clin. Cancer Res..

[29] Ribatti D., Crivellato E. (2009). Immune cells and angiogenesis. J. Cell Mol. Med..

[30] Follo M.Y., Manzoli L., Poli A., McCubrey J.A., Cocco L. (2015). PLC and PI3K/Akt/mTOR signalling in disease and cancer. Adv. Biol. Regul..

[31] Liu Q., Li A., Tian Y., Wu J.D., Liu Y., Li T., Chen Y., Han X., Wu K. (2016). The CXCL8-CXCR1/2 pathways in cancer. Cytokine Growth Factor Rev..

[32] Kemp D.M., Pidich A., Larijani M., Jonas R., Lash E., Sato T., Terai M., De Pizzol M., Allegretti M., Igoucheva O. (2017). Ladarixin, a dual CXCR1/2 inhibitor, attenuates experimental melanomas harboring different molecular defects by affecting malignant cells and tumor microenvironment. Oncotarget.

[33] Moser B., Clark-Lewis I., Zwahlen R., Baggiolini M. (1990). Neutrophil-activating properties of the melanoma growth-stimulatory activity. J. Exp. Med..

[34] Ferrucci P.F., Gandini S., Battaglia A., Alfieri S., Di Giacomo A.M., Giannarelli D., Cappellini G.C., De Galitiis F., Marchetti P., Amato G. (2015). Baseline neutrophil-to-lymphocyte ratio is associated with outcome of ipilimumab-treated metastatic melanoma patients. Br. J. Cancer.

[35] Jensen T.O., Schmidt H., Møller H.J., Donskov F., Høyer M., Sjoegren P., Christensen I.J., Steiniche T. (2012). Intratumoral neutrophils and plasmacytoid dendritic cells indicate poor prognosis and are associated with pSTAT3 expression in AJCC stage I/II melanoma. Cancer.

[36] Masucci M.T., Minopoli M., Carriero M.V. (2019). Tumor Associated Neutrophils. Their Role in Tumorigenesis, Metastasis, Prognosis and Therapy. Front. Oncol..

[37] Jaillon S., Ponzetta A., Di Mitri D., Santoni A., Bonecchi R., Mantovani A. (2020). Neutrophil diversity and plasticity in tumour progression and therapy. Nat. Rev. Cancer.

[38] Verbeke H., Struyf S., Berghmans N., Van Coillie E., Opdenakker G., Uyttenhove C., Van Snick J., Van Damme J. (2011). Isotypic neutralizing antibodies against mouse GCP-2/CXCL6 inhibit melanoma growth and metastasis. Cancer Lett..

[39] Caunt M., Hu L., Tang T., Brooks P.C., Ibrahim S., Karpatkin S. (2006). Growth-regulated oncogene is pivotal in thrombin-induced angiogenesis. Cancer Res..

[40] Keeley E.C., Mehrad B., Strieter R.M. (2008). Chemokines as mediators of neovascularization. Arter. Thromb. Vasc. Biol..

[41] Jobe N.P., Rösel D., Dvořánková B., Kodet O., Lacina L., Mateu R., Smetana K., Brábek J. (2016). Simultaneous blocking of IL-6 and IL-8 is sufficient to fully inhibit CAF-induced human melanoma cell invasiveness. Histochem. Cell Biol..

[42] Soler-Cardona A., Forsthuber A., Lipp K., Ebersberger S., Heinz M., Schossleitner K., Buchberger E., Gröger M., Petzelbauer P., Hoeller C. (2018). CXCL5 Facilitates Melanoma Cell-Neutrophil Interaction and Lymph Node Metastasis. J. Invest. Dermatol..

[43] Toh B., Wang X., Keeble J., Sim W.J., Khoo K., Wong W.C., Kato M., Prevost-Blondel A., Thiery J.P., Abastado J.P. (2011). Mesenchymal transition and dissemination of cancer cells is driven by myeloid-derived suppressor cells infiltrating the primary tumor. PLoS Biol..

[44] Idorn M., Skadborg S.K., Kellermann L., Halldórsdóttir H.R., Holmen Olofsson G., Met Ö., Thor Straten P. (2018). Chemokine receptor engineering of T cells with CXCR2 improves homing towards subcutaneous human melanomas in xenograft mouse model. Oncoimmunology.

[45] Wightman S.C., Uppal A., Pitroda S.P., Ganai S., Burnette B., Stack M., Oshima G., Khan S., Huang X., Posner M.C. (2015). Oncogenic CXCL10 signalling drives metastasis development and poor clinical outcome. Br. J. Cancer.

[46] Spranger S., Dai D., Horton B., Gajewski T.F. (2017). Tumor-Residing Batf3 Dendritic Cells Are Required for Effector T Cell Trafficking and Adoptive T Cell Therapy. Cancer Cell.

[47] Amatschek S., Lucas R., Eger A., Pflueger M., Hundsberger H., Knoll C., Grosse-Kracht S., Schuett W., Koszik F., Maurer D. (2011). CXCL9 induces chemotaxis, chemorepulsion and endothelial barrier disruption through CXCR3-mediated activation of melanoma cells. Br. J. Cancer.

[48] Zhang Y., Xu L., Peng M. (2018). CXCR3 is a prognostic marker and a potential target for patients with solid tumors: A meta-analysis. Onco. Targets Ther..

[49] Mendt M., Cardier J.E. (2017). Activation of the CXCR4 chemokine receptor enhances biological functions associated with B16 melanoma liver metastasis. Melanoma Res..

[50] McConnell A.T., Ellis R., Pathy B., Plummer R., Lovat P.E., O’Boyle G. (2016). The prognostic significance and impact of the CXCR4-CXCR7-CXCL12 axis in primary cutaneous melanoma. Br. J. Dermatol..

[51] Longo-Imedio M.I., Longo N., Treviño I., Lázaro P., Sánchez-Mateos P. (2005). Clinical significance of CXCR3 and CXCR4 expression in primary melanoma. Int. J. Cancer.

[52] Murakami T., Maki W., Cardones A.R., Fang H., Tun Kyi A., Nestle F.O., Hwang S.T. (2002). Expression of CXC chemokine receptor-4 enhances the pulmonary metastatic potential of murine B16 melanoma cells. Cancer Res..

[53] Bartolomé R.A., Ferreiro S., Miquilena-Colina M.E., Martínez-Prats L., Soto-Montenegro M.L., García-Bernal D., Vaquero J.J., Agami R., Delgado R., Desco M. (2009). The chemokine receptor CXCR4 and the metalloproteinase MT1-MMP are mutually required during melanoma metastasis to lungs. Am. J. Pathol..

[54] Kim S.Y., Lee C.H., Midura B.V., Yeung C., Mendoza A., Hong S.H., Ren L., Wong D., Korz W., Merzouk A. (2008). Inhibition of the CXCR4/CXCL12 chemokine pathway reduces the development of murine pulmonary metastases. Clin. Exp. Metastasis.

[55] Alimohammadi M., Rahimi A., Faramarzi F., Alizadeh-Navaei R., Rafiei A. (2021). Overexpression of chemokine receptor CXCR4 predicts lymph node metastatic risk in patients with melanoma: A systematic review and meta-analysis. Cytokine.

[56] André N.D., Silva V.A., Ariza C.B., Watanabe M.A., De Lucca F.L. (2015). In vivo knockdown of CXCR4 using jetPEI/CXCR4 shRNA nanoparticles inhibits the pulmonary metastatic potential of B16-F10 melanoma cells. Mol. Med. Rep..

[57] Blattner C., Fleming V., Weber R., Himmelhan B., Altevogt P., Gebhardt C., Schulze T.J., Razon H., Hawila E., Wildbaum G. (2018). CCR5. Cancer Res..

[58] Umansky V., Blattner C., Gebhardt C., Utikal J. (2017). CCR5 in recruitment and activation of myeloid-derived suppressor cells in melanoma. Cancer Immunol. Immunother..

[59] Weber R., Riester Z., Hüser L., Sticht C., Siebenmorgen A., Groth C., Hu X., Altevogt P., Utikal J.S., Umansky V. (2020). IL-6 regulates CCR5 expression and immunosuppressive capacity of MDSC in murine melanoma. J. Immunother. Cancer.

[60] De Cicco P., Ercolano G., Ianaro A. (2020). The New Era of Cancer Immunotherapy: Targeting Myeloid-Derived Suppressor Cells to Overcome Immune Evasion. Front. Immunol..

[61] Song J.K., Park M.H., Choi D.Y., Yoo H.S., Han S.B., Yoon D.Y., Hong J.T. (2012). Deficiency of C-C chemokine receptor 5 suppresses tumor development via inactivation of NF-κB and upregulation of IL-1Ra in melanoma model. PLoS ONE.

[62] Liu J., Wang C., Ma X., Tian Y., Fu Y., Luo Y. (2019). High expression of CCR5 in melanoma enhances epithelial-mesenchymal transition and metastasis via TGFβ1. J. Pathol..

[63] Cioplea M., Nichita L., Georgescu D., Sticlaru L., Cioroianu A., Nedelcu R., Turcu G., Rauta A., Mogodici C., Zurac S. (2020). FOXP3 in Melanoma with Regression: Between Tumoral Expression and Regulatory T Cell Upregulation. J. Immunol. Res..

[64] Plitas G., Konopacki C., Wu K., Bos P.D., Morrow M., Putintseva E.V., Chudakov D.M., Rudensky A.Y. (2016). Regulatory T Cells Exhibit Distinct Features in Human Breast Cancer. Immunity.

[65] McCully M.L., Moser B. (2011). The human cutaneous chemokine system. Front. Immunol..

[66] Ohue Y., Nishikawa H. (2019). Regulatory T (Treg) cells in cancer: Can Treg cells be a new therapeutic target?. Cancer Sci..

[67] Klarquist J., Tobin K., Farhangi Oskuei P., Henning S.W., Fernandez M.F., Dellacecca E.R., Navarro F.C., Eby J.M., Chatterjee S., Mehrotra S. (2016). Ccl22 Diverts T Regulatory Cells and Controls the Growth of Melanoma. Cancer Res..

[68] Klein A., Sagi-Assif O., Meshel T., Telerman A., Izraely S., Ben-Menachem S., Bayry J., Marzese D.M., Ohe S., Hoon D.S.B. (2017). CCR4 is a determinant of melanoma brain metastasis. Oncotarget.

[69] Förster R., Davalos-Misslitz A.C., Rot A. (2008). CCR7 and its ligands: Balancing immunity and tolerance. Nat. Rev. Immunol..

[70] Schulz O., Hammerschmidt S.I., Moschovakis G.L., Förster R. (2016). Chemokines and Chemokine Receptors in Lymphoid Tissue Dynamics. Annu. Rev. Immunol..

[71] Shields J.D., Emmett M.S., Dunn D.B., Joory K.D., Sage L.M., Rigby H., Mortimer P.S., Orlando A., Levick J.R., Bates D.O. (2007). Chemokine-mediated migration of melanoma cells towards lymphatics—A mechanism contributing to metastasis. Oncogene.

[72] Wiley H.E., Gonzalez E.B., Maki W., Wu M.T., Hwang S.T. (2001). Expression of CC chemokine receptor-7 and regional lymph node metastasis of B16 murine melanoma. J. Natl. Cancer Inst..

[73] Emmett M.S., Lanati S., Dunn D.B., Stone O.A., Bates D.O. (2011). CCR7 mediates directed growth of melanomas towards lymphatics. Microcirculation.

[74] Bedognetti D., Spivey T.L., Zhao Y., Uccellini L., Tomei S., Dudley M.E., Ascierto M.L., De Giorgi V., Liu Q., Delogu L.G. (2013). CXCR3/CCR5 pathways in metastatic melanoma patients treated with adoptive therapy and interleukin-2. Br. J. Cancer.

[75] Yamano T., Kaneda Y., Huang S., Hiramatsu S.H., Hoon D.S. (2006). Enhancement of immunity by a DNA melanoma vaccine against TRP2 with CCL21 as an adjuvant. Mol. Ther..

[76] Chen P., Luo S., Wen Y.J., Li Y.H., Li J., Wang Y.S., Du L.C., Zhang P., Tang J., Yang D.B. (2014). Low-dose paclitaxel improves the therapeutic efficacy of recombinant adenovirus encoding CCL21 chemokine against murine cancer. Cancer Sci..

[77] Adutler-Lieber S., Friedman N., Geiger B. (2018). Expansion and Antitumor Cytotoxicity of T-Cells Are Augmented by Substrate-Bound CCL21 and Intercellular Adhesion Molecule 1. Front. Immunol..

[78] Amersi F.F., Terando A.M., Goto Y., Scolyer R.A., Thompson J.F., Tran A.N., Faries M.B., Morton D.L., Hoon D.S. (2008). Activation of CCR9/CCL25 in cutaneous melanoma mediates preferential metastasis to the small intestine. Clin. Cancer Res..

[79] Homey B., Alenius H., Müller A., Soto H., Bowman E.P., Yuan W., McEvoy L., Lauerma A.I., Assmann T., Bünemann E. (2002). CCL27-CCR10 interactions regulate T cell-mediated skin inflammation. Nat. Med..

[80] Murakami T., Cardones A.R., Finkelstein S.E., Restifo N.P., Klaunberg B.A., Nestle F.O., Castillo S.S., Dennis P.A., Hwang S.T. (2003). Immune evasion by murine melanoma mediated through CC chemokine receptor-10. J. Exp. Med..

[81] Ren T., Chen Q., Tian Z., Wei H. (2007). Down-regulation of surface fractalkine by RNA interference in B16 melanoma reduced tumor growth in mice. Biochem. Biophys. Res. Commun..

[82] Weiss S.A., Han S.W., Lui K., Tchack J., Shapiro R., Berman R., Zhong J., Krogsgaard M., Osman I., Darvishian F. (2016). Immunologic heterogeneity of tumor-infiltrating lymphocyte composition in primary melanoma. Hum. Pathol..

[83] López-Janeiro Á., Padilla-Ansala C., de Andrea C.E., Hardisson D., Melero I. (2020). Prognostic value of macrophage polarization markers in epithelial neoplasms and melanoma. A systematic review and meta-analysis. Mod. Pathol..

[84] Robinson A.V., Keeble C., Lo M.C.I., Thornton O., Peach H., Moncrieff M.D.S., Dewar D.J., Wade R.G. (2020). The neutrophil-lymphocyte ratio and locoregional melanoma: A multicentre cohort study. Cancer Immunol. Immunother..

[85] Iacono D., Basile D., Gerratana L., Vitale M.G., Pelizzari G., Cinausero M., Poletto E., Puglisi F., Fasola G., Minisini A.M. (2019). Prognostic role of disease extent and lymphocyte-monocyte ratio in advanced melanoma. Melanoma. Res..

[86] Loetscher P., Uguccioni M., Bordoli L., Baggiolini M., Moser B., Chizzolini C., Dayer J.M. (1998). CCR5 is characteristic of Th1 lymphocytes. Nature.

[87] De Simone M., Arrigoni A., Rossetti G., Gruarin P., Ranzani V., Politano C., Bonnal R.J.P., Provasi E., Sarnicola M.L., Panzeri I. (2016). Transcriptional Landscape of Human Tissue Lymphocytes Unveils Uniqueness of Tumor-Infiltrating T Regulatory Cells. Immunity.

[88] Doi T., Muro K., Ishii H., Kato T., Tsushima T., Takenoyama M., Oizumi S., Gemmoto K., Suna H., Enokitani K. (2019). A Phase I Study of the Anti-CC Chemokine Receptor 4 Antibody, Mogamulizumab, in Combination with Nivolumab in Patients with Advanced or Metastatic Solid Tumors. Clin. Cancer Res..

[89] Ureshino H., Shindo T., Nishikawa H., Watanabe N., Watanabe E., Satoh N., Kitaura K., Kitamura H., Doi K., Nagase K. (2016). Effector Regulatory T Cells Reflect the Equilibrium between Antitumor Immunity and Autoimmunity in Adult T-cell Leukemia. Cancer Immunol. Res..

[90] Van Damme H., Dombrecht B., Kiss M., Roose H., Allen E., Van Overmeire E., Kancheva D., Martens L., Murgaski A., Bardet P.M.R. (2021). Therapeutic depletion of CCR8. J. Immunother. Cancer.

[91] Whiteside S.K., Grant F.M., Gyori D.S., Conti A.G., Imianowski C.J., Kuo P., Nasrallah R., Sadiyah F., Lira S.A., Tacke F. (2021). CCR8 marks highly suppressive Treg cells within tumours but is dispensable for their accumulation and suppressive function. Immunology.

[92] Cho W.C., Jour G., Aung P.P. (2019). Role of angiogenesis in melanoma progression: Update on key angiogenic mechanisms and other associated components. Semin. Cancer Biol..

[93] Wang T., Xiao M., Ge Y., Krepler C., Belser E., Lopez-Coral A., Xu X., Zhang G., Azuma R., Liu Q. (2015). BRAF Inhibition Stimulates Melanoma-Associated Macrophages to Drive Tumor Growth. Clin. Cancer Res..

[94] Liu G., Zhang F., Lee J., Dong Z. (2005). Selective induction of interleukin-8 expression in metastatic melanoma cells by transforming growth factor-beta 1. Cytokine.

[95] Torisu H., Ono M., Kiryu H., Furue M., Ohmoto Y., Nakayama J., Nishioka Y., Sone S., Kuwano M. (2000). Macrophage infiltration correlates with tumor stage and angiogenesis in human malignant melanoma: Possible involvement of TNFalpha and IL-1alpha. Int. J. Cancer.

[96] Hanahan D., Weinberg R.A. (2000). The hallmarks of cancer. Cell.

[97] Chambers A.F., Groom A.C., MacDonald I.C. (2002). Dissemination and growth of cancer cells in metastatic sites. Nat. Rev. Cancer.

[98] Marconi C., Bianchini F., Mannini A., Mugnai G., Ruggieri S., Calorini L. (2008). Tumoral and macrophage uPAR and MMP-9 contribute to the invasiveness of B16 murine melanoma cells. Clin. Exp. Metastasis.

[99] Pastushenko I., Vermeulen P.B., Van den Eynden G.G., Rutten A., Carapeto F.J., Dirix L.Y., Van Laere S. (2014). Mechanisms of tumour vascularization in cutaneous malignant melanoma: Clinical implications. Br. J. Dermatol..

[100] Harlin H., Meng Y., Peterson A.C., Zha Y., Tretiakova M., Slingluff C., McKee M., Gajewski T.F. (2009). Chemokine expression in melanoma metastases associated with CD8+ T-cell recruitment. Cancer Res..

[101] Hodorogea A., Calinescu A., Antohe M., Balaban M., Nedelcu R.I., Turcu G., Ion D.A., Badarau I.A., Popescu C.M., Popescu R. (2019). Epithelial-Mesenchymal Transition in Skin Cancers: A Review. Anal. Cell Pathol..

[102] Zhou S.L., Zhou Z.J., Hu Z.Q., Li X., Huang X.W., Wang Z., Fan J., Dai Z., Zhou J. (2015). CXCR2/CXCL5 axis contributes to epithelial-mesenchymal transition of HCC cells through activating PI3K/Akt/GSK-3β/Snail signaling. Cancer Lett..

[103] Zhao J., Ou B., Han D., Wang P., Zong Y., Zhu C., Liu D., Zheng M., Sun J., Feng H. (2017). Tumor-derived CXCL5 promotes human colorectal cancer metastasis through activation of the ERK/Elk-1/Snail and AKT/GSK3β/β-catenin pathways. Mol. Cancer.

[104] Mao Z., Zhang J., Shi Y., Li W., Shi H., Ji R., Mao F., Qian H., Xu W., Zhang X. (2020). CXCL5 promotes gastric cancer metastasis by inducing epithelial-mesenchymal transition and activating neutrophils. Oncogenesis.

[105] Naxerova K., Reiter J.G., Brachtel E., Lennerz J.K., van de Wetering M., Rowan A., Cai T., Clevers H., Swanton C., Nowak M.A. (2017). Origins of lymphatic and distant metastases in human colorectal cancer. Science.

[106] Luo B.H., Carman C.V., Springer T.A. (2007). Structural basis of integrin regulation and signaling. Annu. Rev. Immunol..

[107] Cardones A.R., Murakami T., Hwang S.T. (2003). CXCR4 enhances adhesion of B16 tumor cells to endothelial cells in vitro and in vivo via beta(1) integrin. Cancer Res..

[108] Haqq C., Nosrati M., Sudilovsky D., Crothers J., Khodabakhsh D., Pulliam B.L., Federman S., Miller J.R., Allen R.E., Singer M.I. (2005). The gene expression signatures of melanoma progression. Proc. Natl. Acad. Sci. USA.

[109] Mitchell B., Leone D., Feller J.K., Bondzie P., Yang S., Park H.Y., Mahalingam M. (2014). Correlation of chemokine receptor CXCR4 mRNA in primary cutaneous melanoma with established histopathologic prognosticators and the BRAF status. Melanoma. Res..

[110] Mitchell B., Leone D., Feller K., Menon S., Bondzie P., Yang S., Park H.Y., Mahalingam M. (2014). Protein expression of the chemokine receptor CXCR4 and its ligand CXCL12 in primary cutaneous melanoma--biomarkers of potential utility?. Hum. Pathol..

[111] Ortega-Bernal D., La Rosa C.H.G., Arechaga-Ocampo E., Alvarez-Avitia M.A., Moreno N.S., Rangel-Escareño C. (2018). A meta-analysis of transcriptome datasets characterizes malignant transformation from melanocytes and nevi to melanoma. Oncol. Lett..

[112] Martinez-Rodriguez M., Thompson A.K., Monteagudo C. (2017). High CCL27 immunoreactivity in ’supratumoral’ epidermis correlates with better prognosis in patients with cutaneous malignant melanoma. J. Clin. Pathol..

[113] Simonetti O., Goteri G., Lucarini G., Filosa A., Pieramici T., Rubini C., Biagini G., Offidani A. (2006). Potential role of CCL27 and CCR10 expression in melanoma progression and immune escape. Eur. J. Cancer.

[114] Cesati M., Scatozza F., D’Arcangelo D., Antonini-Cappellini G.C., Rossi S., Tabolacci C., Nudo M., Palese E., Lembo L., Di Lella G. (2020). Investigating Serum and Tissue Expression Identified a Cytokine/Chemokine Signature as a Highly Effective Melanoma Marker. Cancers.

[115] Zhou X., Peng M., He Y., Peng J., Zhang X., Wang C., Xia X., Song W. (2021). CXC Chemokines as Therapeutic Targets and Prognostic Biomarkers in Skin Cutaneous Melanoma Microenvironment. Front. Oncol..

[116] Fujimura T., Sato Y., Tanita K., Lyu C., Kambayashi Y., Amagai R., Otsuka A., Fujisawa Y., Yoshino K., Matsushita S. (2019). Association of Baseline Serum Levels of CXCL5 With the Efficacy of Nivolumab in Advanced Melanoma. Front. Med..

[117] Sanmamed M.F., Perez-Gracia J.L., Schalper K.A., Fusco J.P., Gonzalez A., Rodriguez-Ruiz M.E., Oñate C., Perez G., Alfaro C., Martín-Algarra S. (2017). Changes in serum interleukin-8 (IL-8) levels reflect and predict response to anti-PD-1 treatment in melanoma and non-small-cell lung cancer patients. Ann. Oncol..

[118] Ugurel S., Rappl G., Tilgen W., Reinhold U. (2001). Increased serum concentration of angiogenic factors in malignant melanoma patients correlates with tumor progression and survival. J. Clin. Oncol..

[119] Sanmamed M.F., Carranza-Rua O., Alfaro C., Oñate C., Martín-Algarra S., Perez G., Landazuri S.F., Gonzalez A., Gross S., Rodriguez I. (2014). Serum interleukin-8 reflects tumor burden and treatment response across malignancies of multiple tissue origins. Clin. Cancer Res..

[120] Lok E., Chung A.S., Swanson K.D., Wong E.T. (2014). Melanoma brain metastasis globally reconfigures chemokine and cytokine profiles in patient cerebrospinal fluid. Melanoma. Res..

[121] Tabolacci C., Cordella M., Mariotti S., Rossi S., Senatore C., Lintas C., Levati L., D’Arcangelo D., Facchiano A., D’Atri S. (2021). Melanoma Cell Resistance to Vemurafenib Modifies Inter-Cellular Communication Signals. Biomedicines.

[122] Knight D.A., Ngiow S.F., Li M., Parmenter T., Mok S., Cass A., Haynes N.M., Kinross K., Yagita H., Koya R.C. (2013). Host immunity contributes to the anti-melanoma activity of BRAF inhibitors. J. Clin. Invest..

[123] Bellmann L., Cappellano G., Schachtl-Riess J.F., Prokopi A., Seretis A., Ortner D., Tripp C.H., Brinckerhoff C.E., Mullins D.W., Stoitzner P. (2020). A TLR7 agonist strengthens T and NK cell function during BRAF-targeted therapy in a preclinical melanoma model. Int. J. Cancer.

[124] Simon B., Uslu U. (2018). CAR-T cell therapy in melanoma: A future success story?. Exp. Dermatol..

[125] Idorn M., Olsen M., Halldórsdóttir H.R., Skadborg S.K., Pedersen M., Høgdall C., Høgdall E., Met Ö., Thor Straten P. (2018). Improved migration of tumor ascites lymphocytes to ovarian cancer microenvironment by CXCR2 transduction. Oncoimmunology.

[126] Mikucki M.E., Fisher D.T., Matsuzaki J., Skitzki J.J., Gaulin N.B., Muhitch J.B., Ku A.W., Frelinger J.G., Odunsi K., Gajewski T.F. (2015). Non-redundant requirement for CXCR3 signalling during tumoricidal T-cell trafficking across tumour vascular checkpoints. Nat. Commun..

[127] Wennerberg E., Kremer V., Childs R., Lundqvist A. (2015). CXCL10-induced migration of adoptively transferred human natural killer cells toward solid tumors causes regression of tumor growth in vivo. Cancer Immunol. Immunother..

[128] Antonicelli F., Lorin J., Kurdykowski S., Gangloff S.C., Le Naour R., Sallenave J.M., Hornebeck W., Grange F., Bernard P. (2011). CXCL10 reduces melanoma proliferation and invasiveness in vitro and in vivo. Br. J. Dermatol..

[129] D’Alterio C., Buoncervello M., Ieranò C., Napolitano M., Portella L., Rea G., Barbieri A., Luciano A., Scognamiglio G., Tatangelo F. (2019). Targeting CXCR4 potentiates anti-PD-1 efficacy modifying the tumor microenvironment and inhibiting neoplastic PD-1. J. Exp. Clin. Cancer Res..

[130] Wetzel K., Struyf S., Van Damme J., Kayser T., Vecchi A., Sozzani S., Rommelaere J., Cornelis J.J., Dinsart C. (2007). MCP-3 (CCL7) delivered by parvovirus MVMp reduces tumorigenicity of mouse melanoma cells through activation of T lymphocytes and NK cells. Int. J. Cancer.

[131] Salerno E.P., Olson W.C., McSkimming C., Shea S., Slingluff C.L. (2014). T cells in the human metastatic melanoma microenvironment express site-specific homing receptors and retention integrins. Int. J. Cancer.

[132] Matsuo K., Itoh T., Koyama A., Imamura R., Kawai S., Nishiwaki K., Oiso N., Kawada A., Yoshie O., Nakayama T. (2016). CCR4 is critically involved in effective antitumor immunity in mice bearing intradermal B16 melanoma. Cancer Lett..

[133] Mestas J., Hughes C.C. (2004). Of mice and not men: Differences between mouse and human immunology. J. Immunol..

[134] Mehrad B., Keane M.P., Strieter R.M. (2007). Chemokines as mediators of angiogenesis. Thromb. Haemost..

[135] Kim M., Koh Y.J., Kim K.E., Koh B.I., Nam D.H., Alitalo K., Kim I., Koh G.Y. (2010). CXCR4 signaling regulates metastasis of chemoresistant melanoma cells by a lymphatic metastatic niche. Cancer Res..

[136] Lee N., Barthel S.R., Schatton T. (2014). Melanoma stem cells and metastasis: Mimicking hematopoietic cell trafficking?. Lab. Investig..

[137] Saxena S., Singh R.K. (2021). Chemokines orchestrate tumor cells and the microenvironment to achieve metastatic heterogeneity. Cancer Metastasis Rev..

